# Adolescent Health Promotion Interventions Using Well-Care Visits and a Smartphone Cognitive Behavioral Therapy App: Randomized Controlled Trial

**DOI:** 10.2196/34154

**Published:** 2022-05-23

**Authors:** Shinichiro Nagamitsu, Ayako Kanie, Kazumi Sakashita, Ryoichi Sakuta, Ayumi Okada, Kencho Matsuura, Masaya Ito, Akiko Katayanagi, Takashi Katayama, Ryoko Otani, Tasuku Kitajima, Naoki Matsubara, Takeshi Inoue, Chie Tanaka, Chikako Fujii, Yoshie Shigeyasu, Ryuta Ishii, Sayaka Sakai, Michiko Matsuoka, Tatsuyuki Kakuma, Yushiro Yamashita, Masaru Horikoshi

**Affiliations:** 1 Department of Pediatrics Fukuoka University Faculty of Medicine Fukuoka Japan; 2 Department of Pediatrics and Child Health Kurume University School of Medicine Kurume Japan; 3 National Center for Cognitive Behavior Therapy and Research National Center of Neurology and Psychiatry Kodaira Japan; 4 Department of Interdisciplinary Medicine National Center for Child Health and Development Setagaya Japan; 5 Child Development and Psychosomatic Medicine Center Dokkyo Medical University Saitama Medical Center Koshigaya Japan; 6 Department of Pediatrics Okayama University Graduate School of Medicine Dentistry and Pharmaceutical Sciences Okayama Japan; 7 Department of Nursing Fukuoka Prefectural University Tagawa Japan; 8 Life2Bits Shibuya Japan; 9 Department of Neuropsychiatry Kurume University School of Medicine Kurume Japan; 10 Biostatistics Center Kurume University Kurume Japan

**Keywords:** health promotion, well-care visit, cognitive behavioral therapy, app, randomized controlled trial, RCT, mobile phone

## Abstract

**Background:**

Adolescent health promotion is important in preventing risk behaviors and improving mental health. Health promotion during adolescence has been shown to contribute to the prevention of late onset of the mental health disease. However, scalable interventions have not been established yet.

**Objective:**

This study was designed to test the efficacy of two adolescent health promotion interventions: a well-care visit (WCV) with a risk assessment interview and counseling and self-monitoring with a smartphone cognitive behavioral therapy (CBT) app. Our hypothesis was that participants who had received both WCV and the CBT app would have better outcomes than those who had received only WCV or those who had not received any intervention. We conducted a prospective multi-institutional randomized controlled trial.

**Methods:**

Participants were 217 adolescents aged 13-18 years. They were randomly divided into two intervention groups (WCV group and WCV with CBT app group) and a nonintervention group. WCV comprised a standardized physical examination along with a structured interview and counseling for youth risk assessment, which was designed with reference to the Guideline for Health Supervision of Adolescents of Bright Futures. A smartphone-based CBT program was developed based on the CBT approach. The CBT app comprised a 1-week psychoeducation component and a 1-week self-monitoring component. During the CBT program, participants created several self-monitoring sheets based on the CBT model with five window panels: event, thoughts, feelings, body response, and actions. The primary outcome was the change in scores for depressive symptoms. Secondary outcomes included changes in scores for self-esteem, quality of life, self-monitoring, and an adolescent health promotion scale. These outcomes were evaluated at baseline and at 1, 2, and 4 months after baseline. The exploratory outcome was the presence of suicidal ideation during the observation period. Intervention effects were estimated using mixed effect models.

**Results:**

In total, 94% (204/217) of the participants completed the 4-month evaluation. Both intervention groups showed a significant effect in the form of reduced scores for depressive symptoms at 1 month in high school students; however, these effects were not observed at 2 and 4 months. The intervention effect was significantly more predominant in those scoring above cutoff for depressive symptoms. There was significantly less suicidal ideation in the intervention groups. As for secondary outcomes, there was significant increase in health promotion scale scores at the 4-month follow-up among junior high school students in the WCV group. Moreover, the CBT app was significantly effective in terms of obtaining self-monitoring skills and reducing depressive symptoms.

**Conclusions:**

Although adolescent health promotion interventions may have short-term benefits, the frequency of WCV and further revision of the CBT app should be considered to evaluate long-term effectiveness.

**Trial Registration:**

University Hospital Medical Information Network Clinical Trials Registry UMIN 000036343; https://center6.umin.ac.jp/cgi-open-bin/ctr/ctr_view.cgi?recptno=R000041246

## Introduction

### Background

Care settings for young children and adolescents are changing from treating acute diseases to managing chronic diseases as vaccinations are developed and deployed and as medical treatment advances. With reductions in serious illnesses, physicians can place greater importance on lifestyle-related diseases, mental health disorders, and developmental behavioral disorders [1,2]. The Ministry of Health, Labour and Welfare, Japan, published its first report concerning disability-adjusted life years of Japanese adolescents in 2018, which indicated that mental health disorders accounted for approximately 20% of the burden of disease [3]. Mental health disorders such as depression affect approximately 5% of adolescents in Japan, and suicide is the leading cause of death among adolescents [4]. Therefore, health supervision for young children and adolescents is becoming increasingly important in medical settings.

### Primary Care Visits

The American Academy of Pediatrics recommended the delivery of preventive services and anticipatory guidance for adolescents aged ≤21 years through annual primary care visits [5,6]. These visits offer an important opportunity that may lead to reduced risk behaviors among adolescents [7,8]. Evidence shows that although preventive interventions resulted in various significant improvements such as reduced smoking, increased helmet use, and increased condom use, there are insufficient effects in terms of reduced substance and alcohol use and change in the rate of sexual intercourse [9,10]. Furthermore, the screening rate for major depression disorder (MDD) among adolescents is insufficient in the context of annual primary care visits [11]. The low MDD screening rate may result from insufficient training of pediatric health care providers. Fallucco et al [12] reported that the MDD screening rate significantly increased after primary care providers received adequate training in depression care. As the primary care visit rate among adolescents is <50%, it is important to provide opportunities for routine visits to reduce risk behaviors and promote health in this population.

### Internet-Based Cognitive Behavioral Therapy

A universal intervention program based on cognitive behavioral therapy (CBT) using an internet-based setting has shown potential to prevent depressive symptoms among adolescents [13-19]. These intervention techniques have been proposed as self-help strategies to relieve depressive symptoms for adolescents, as young children and adolescents with depressive symptoms often do not receive medical treatment owing to lack of symptom awareness, poor access to services, and perceived stigma [20]. Several randomized controlled trials have demonstrated the efficacy of internet-based therapies for depression. Moritz et al [13] reported an 8-week internet-based program that encompassed 10 content modules focused on evidence-based cognitive behavioral techniques (eg, psychoeducation, behavioral activation, and problem solving) and showed a significant decline in symptoms of depression in adulthood. However, the efficacy of internet-based CBT for adolescent depression remains inconclusive. Pennant et al [18] systematically reviewed the evidence for internet-based CBT interventions for adolescents and showed a small positive effect for depression in a general population study. Kauer et al [15] reported that an internet-based CBT intervention for adolescents had a significant effect on depressive symptoms. The differences in study findings may be attributable to the duration and strength of the intervention, contamination effect, degree of depressive symptoms, and amount of guidance provided to the participants.

### Objectives

Both primary care visits and internet-based CBT programs may be beneficial in promoting adolescent health. Interestingly, these intervention procedures are delivered in completely opposite ways; the former is characterized by a face-to-face encounter, whereas the latter is based on self-help therapy without an interview. Face-to-face encounters have the advantage of providing health education securely and allow providers to respond to individual requirements, but have disadvantages in terms of cost and time. Although internet-based programs have various advantages such as accessibility to large groups, cost-effectiveness, and less labor, a major disadvantage is that they depend on user motivation. In this study, we conduct a randomized controlled trial with a well-care visit (WCV) combined with a risk assessment interview and counseling and self-monitoring using a smartphone CBT app to promote adolescent health. The primary outcome of the interventions is improvement in depressive symptoms, and the secondary outcome is increase in health promotion score. Our hypothesis is that participants who receive both WCV and the CBT app would have better outcomes than those who receive only WCV or those who did not receive any intervention.

## Methods

### Study Design

**Figure 1:**
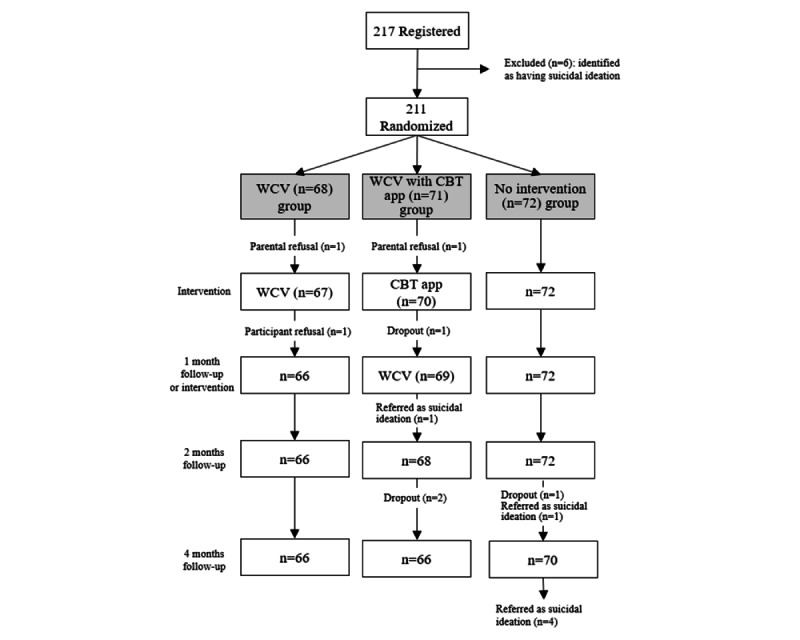
Participant flow chart. CBT: cognitive behavioral therapy; WCV: well-care visit.

We conducted a prospective multi-institutional randomized controlled trial involving 217 adolescents (aged 13-18 years) from Fukuoka, Saitama, and Okayama prefectures and Tokyo. The trial was registered in the University Hospital Medical Information Network Clinical Trials Registry (UMIN 000036343). Participants were enrolled in the trial and randomized into two intervention groups (WCV only or WCV with CBT app) or a nonintervention (control) group. Outcome data were collected at baseline, after the intervention (4 weeks), and at 2- and 4-month follow-ups. Figure 1 shows a participant flow chart demonstrating participant allocation, intervention menu, and data collection. Participants in the nonintervention group were offered the intervention immediately after this study was completed.

### Ethics Approval

The design of this study and procedures for obtaining informed consent were approved by the Medical Ethics Committee of the Kurume University School of Medicine (#18138).

### Procedure

The principal investigator and coinvestigators in each prefecture explained the purpose of this study and the study design to each regional educational committee and school principals’ association. School principals who were interested in the study informed students in their school about the content of this study using a leaflet. The leaflet was developed by the principal investigator and covered the purpose of the study, study design, participant recruitment, and the URL for the study’s home page. Students who were interested in this study could talk with their parents about enrollment. To receive a detailed explanation of the study design, the student or their parents applied for an appointment with an investigator at the relevant research facility (hospital outpatient clinic) through the study’s home page. During these appointments, students and their parents received detailed information about the study and provided informed consent. A total of 240 students from 23 junior high schools and 25 high schools received appointments, and 217 (90.4%) of them agreed to participate in this study. The inclusion criteria were the following: (1) aged 13-18 years, (2) able to visit a research facility with their parent or caregiver twice to receive a WCV or for installation of the CBT program, and (3) had access to a smartphone or Wi-Fi network (smartphones were available to rent if a participant had no smartphone). The exclusion criterion was the presence of severe depressive symptoms or suicidal ideation. After the participants and parents signed the informed consent form, screening for severe depressive symptoms or suicidal ideation was performed using the 9-item Patient Health Questionnaire (PHQ-9) [21]. A PHQ-9 item asks how often responders had thoughts of hurting themselves or thoughts that it would be better if they were dead over the past 2 weeks. For individuals who scored 2 or 3 (“more than half” or “almost every day,” respectively), participation was suspended before randomization, and they were referred to mental health services. Group allocation was stratified by sex and school type (junior high school or high school). A research assistant, who was not otherwise engaged in this study, generated the random dynamic allocation sequence using a minimization method. After each participant provided informed consent, they were automatically allocated to an intervention group or the nonintervention group within 2 weeks. Allocation was concealed from the principal investigator and all coinvestigators dealing with the participants.

### Interventions

#### Overview

This study had two intervention groups (WCV group and WCV with CBT app group) and a nonintervention group. Participants in all groups were asked to complete a questionnaire that included several outcome measures at four time points: at baseline and at 1, 2, and 4 months after baseline. The participant flow chart is shown in Figure 1. Participants in the WCV group visited the research facility twice (at baseline and 1 month after their first visit) to undergo a health checkup along with a risk assessment interview and counseling. They completed the baseline and 1-month outcome questionnaires before the respective visit to the research facility. The questionnaires for the 2- and 4-month measurements were delivered to the participants’ homes, and completed questionnaires were returned to the respective research facility. Participants in the WCV with CBT app group visited a research facility twice (at baseline and 1 month after the first visit). During the first visit, the participants completed the baseline questionnaire and installed the CBT app program on their smartphone. As the CBT app involved a 2-week program, participants had a second visit after 1 month to receive their health checkup. These participants were required to complete the smartphone CBT program before undergoing their health checkup. They completed the baseline and 1-month questionnaires at the research facility and the 2- and 4-month questionnaires at their homes. For the nonintervention group, the questionnaires were delivered to participants’ homes at each time point and the completed questionnaires were returned. After the intervention period (4 months), participants in the nonintervention group received either WCV or the CBT app (or both) as needed.

#### Contents of WCV

The WCV was designed with reference to the Guideline for Health Supervision of Adolescents of Bright Futures. The purpose of the WCV was to address the individual’s concerns or stressors, check social determinants of health, and provide anticipatory guidance through an interview. We used the Home, Education, Eating, Activities, Drugs, Sexuality, Suicide, Safety (HEEADSSS) framework to help structure the WCV interviews [22]. A complete physical examination was also included in the WCV, which involves measuring blood pressure, height, and weight; checking BMI, scoliosis, and acne; and rating sexual maturity. Before the WCV, participants were asked to complete the outcome questionnaire. The WCV consisted of a 40-minute session: checking individual history and the participant’s concerns or stressors using check sheets (5 minutes), risk assessment interview using the HEEADSSS framework (20 minutes), physical examination (5 minutes), and discussing anticipatory guidance (10 minutes). Guidance was also provided to participants’ parents or guardians, as needed. After the individual risk assessment, participants received educational handouts that described how to avoid and manage risk behaviors. We prepared 20 different handouts, covering the following aspects: sleep hygiene, appropriate eating, dieting, obesity, screen time, exercising, headache, oral health, constipation, acne, menstruation, sports injury, helmets or seat belts, school record, relationships with friends, mental health, tobacco, alcohol, sexual behavior, and the internet. During the second WCV, the participants discussed issues that had been determined during their first WCV. For example, if prolonged screen time was noted during the first WCV, the participant’s effort to improve this was discussed during the second WCV. To standardize the WCV procedure among research facilities, all the investigators providing WCVs received training using a demonstration video developed by one of the coauthors, and then, all the investigators gathered at 1 research facility and were further trained through role-play.

#### CBT App

**Figure 2:**
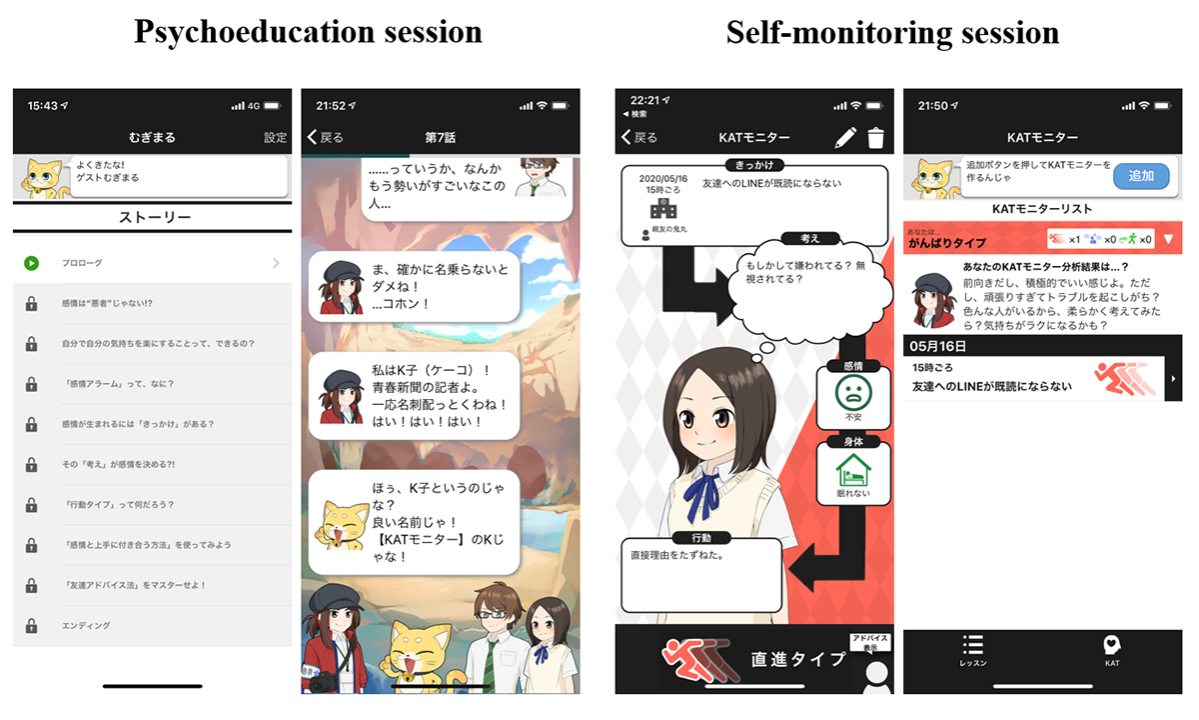
Screenshots of the smartphone cognitive behavioral therapy app.

A smartphone-based CBT program for iPhones, named *Mugimaru*, was developed based on the CBT approach. The program comprised a psychoeducation session (week 1) and a self-monitoring session (week 2). Mugimaru presented psychoeducation in a story-like manner, so that the adolescents can easily understand the rationale of CBT and were motivated to continue using the app. The story featured an adolescent boy, an adolescent girl, and a cat (the name of this cat is *Mugimaru*). In the story, the boy and girl have troubles in relationships with friends or about their future. Mugimaru teaches them how the feelings, thoughts, and actions are mutually affected. They also learn that their feelings are associated with their thoughts and actions. The story consisted of 10 scenarios, and participants could browse 1 to 2 scenarios each day. After reading one scenario, a new scenario could be read after 24 hours. The ending of the story was available 1 week after the participants read the whole story. During the intervention period, participants created several self-monitoring sheets based on the CBT model with five window panels: event, thoughts, feelings, body response, and actions. The participant inputted their thoughts, feelings, body responses, and actions when they experienced a daily event. In another window, the adolescents could input comments or advice if their friend had experienced the same event. This input was used by adolescents to practice cognitive reappraisal and problem solving. Figure 2 shows the CBT app screenshots from the smartphone. By repeatedly creating these monitoring sheets, the adolescents could monitor their own experiences and develop solutions to make necessary changes. The shortest time in which Mugimaru can be completed was 2 weeks. All the data were stored in the main server, and the participants were informed in advance that only the principal investigator could view the data.

#### Depression Self-Rating Scale for Children

The primary outcome of this intervention was improvement in depressive symptoms. The Depression Self-Rating Scale for Children (DSRS-C), an 18-item self-report questionnaire that measures depressive symptoms, was used to measure depressive symptoms in this study [23]. Participants are asked to select one of three response options: “most of the time” (score=2), “sometimes” (score=1), or “never” (score=0). The maximum score is 36, and higher scores indicate stronger depressive tendencies. The Japanese version of the DSRS-C has good reliability and validity [24]. The cutoff score for the Japanese version is 16 points.

#### Adolescent Health Promotion Short Form

The Adolescent Health Promotion Short Form (AHP-SF) is a self-administered instrument that was designed by Chen et al [25] to measure adolescent health-promoting behaviors. The instrument uses a 5-point Likert scale to obtain data regarding the frequency of reported behaviors. Scores range from 1 (“never”) to 5 (“always”). The AHP-SF has 21 items on six subscales: nutrition, social support, health responsibility, life appreciation, exercise, and stress management. The total score ranges from 21 to 105. We obtained permission from the original authors to develop a Japanese version of the AHP-SF.

#### Rosenberg Self-Esteem Scale

The Rosenberg Self-Esteem Scale (RSES) is the most recognized and widely used measure to quantify global positive and negative attitudes toward the self [26]. It comprises 10 items with responses on a 4-point Likert scale: “strongly agree” (score=4), “agree” (score=3), “disagree” (score=2), and “strongly disagree” (score=1). Negatively worded items are reverse scored, and total score ranges from 10 to 40. Higher scores reflect greater levels of self-esteem. The Japanese version of the RSES has good reliability and validity [27].

#### Pediatric Quality of Life Inventory

The Pediatric Quality of Life Inventory (PedsQL) is a brief measure of adolescents’ health-related quality of life [28]. The 23 items comprise four generic core scales: physical functioning, emotional functioning, social functioning, and school functioning. Items are scored as 0 (“never”; score=100), 1 (“almost never”; score=75), 2 (“sometimes”; score=50), 3 (“often”; score=25), and 4 (“almost always”; score=0). The total scale score is calculated from the mean of all the items and transformed to a 0-100 scale. Higher scores indicate better health-related quality of life. The Japanese version of the PedsQL has good reliability and validity [29].

### Exploratory Outcome Measures

#### Suicidal Ideation

We counted the number of participants presenting suicidal ideation on the PHQ-9 during the observation period in each group. We defined participants as having suicidal ideation if they scored 2 or 3 (“more than half” or “almost every day,” respectively) on item 9 of the PHQ-9 [21].

#### Trait Emotional Intelligence Questionnaire–Adolescent Short Form

The Trait Emotional Intelligence Questionnaire–Adolescent Short Form (TEIQue-ASF) assesses how adolescents perceive their ability to deal with their emotions while communicating with others [30,31]. The TEIQue-ASF has 30 items with responses on a 7-point Likert scale, from “strongly disagree” (score=1) to “strongly agree” (score=7). Some items, such as “I’m usually able to find ways to control my emotions when I want to” and “On the whole, I’m able to deal with stress,” from the self-control subscale were used to assess the efficacy of the CBT app.

#### Participants’ Use of the CBT App and the Number of Worksheets Created in the CBT App

Participants’ use of the CBT app was confirmed using server data on the number of days they browsed the CBT app (Mugimaru) and the number of self-monitoring sheets they created during the CBT app intervention period.

### Sample Size Consideration

The sample size for this study was calculated based on the results from previous studies that set depressive symptoms as the primary outcome [13,19,32-34]. We estimated that approximately 75 participants were required to detect group differences in the DSRS-C (mean difference 2.8, SD 6) at 1 month, with 80% power at *P*=.05.

### Data Analysis

#### Primary and Secondary Outcome Measures

To investigate the effect of the intervention on the primary outcome measure (depressive symptoms), data analysis was performed using 2 strategies. First, we assessed changes in the mean depressive scores from baseline to the 1-month evaluation and from baseline to the 4-month evaluation as the immediate effect and maintenance effect of the intervention, respectively. Changes were statistically compared among the WCV, WCV with CBT app, and nonintervention groups using mixed effect models, accounting for the within-participant serial correlation of repeated measures. We also investigated the intervention effect separately for junior high school students and high school students. Second, we examined the associations between baseline depressive symptoms and the intervention. Therefore, participants in both intervention groups were classified into a group with baseline DSRS-C score >16 and another group with baseline DSRS-C score ≤16. An analysis similar to that described above was used to examine the immediate and maintenance effects of the intervention. The nonintervention group was excluded from this assessment. For the secondary outcome measures (including AHP-SF, RSES, and PedsQL scores), the changes in each score from baseline to the 1-, 2-, and 4-month evaluations were compared among the 3 groups using mixed effect models.

Furthermore, to assess the effect of the CBT app on depressive scores, the association between the number of self-monitoring sheets created by participants on their smartphone and the changes in depressive scores at the 1-, 2-, and 4-month evaluations were investigated using correlation coefficients. Similarly, to clarify the self-monitoring efficacy of the CBT app, we assessed the changes in TEIQue-ASF scores from baseline to the 1-, 2-, and 4-month evaluations.

#### Exploratory Outcome Measures

At every measurement point, the presence of suicidal ideation in participants was assessed using PHQ-9. The prevalence of suicidal ideation between the intervention groups and nonintervention group was compared using chi-square test.

As this study was an exploratory investigation of the proposed intervention, no adjustment was used in multiple comparisons. All data analyses were performed using SAS (version 9.4; SAS Institute Inc).

## Results

### Participants and Follow-up Rate

A total of 217 participants from 48 schools (23 junior high schools and 25 high schools) were enrolled in this study and randomized into the 3 groups. From the 217 participants, 6 (2.7%) participants were excluded owing to the presence of suicidal ideation. Thus, 97.2% (211/217) of the participants were included in our analyses (WCV group: 68/211, 32.2%; WCV with CBT app group: 71/211, 33.6%; and nonintervention group: 72/211, 34.1%). There were 37.9% (80/211) male participants and 62.1% (131/211) female participants, with 38.9% (82/211) of the participants from junior high school and 61.1% (129/211) of the participants from high school. During the follow-up period, 1.4% (3/211) of the participants (3/3, 100% women; 2/3, 67% from the WCV group; and 1/3, 33% from the WCV with CBT app group) canceled their study attendance, and 1.9% (4/211) of the participants (1/4, 25% men and 3/4, 75% women; 3/4, 75% from the WCV with CBT app group and 1/4, 25% from the nonintervention group) dropped out of the study without giving any reason. Consequently, the follow-up rate was 96.7% (204/211). The flow of participants is shown in Figure 1.

### DSRS-C Scores

For all participants, the mean changes in DSRS-C scores from baseline to 1, 2, and 4 months did not significantly differ among the WCV group, WCV with CBT app group, and nonintervention group (Table 1).

**Table 1:** Continuous outcome scores from baseline to the follow-up period for each group.

Outcome measure and follow-up (months)		WCV^a^ group (n=68)				WCV with CBT^b^ app group (n=71)				Nonintervention group (n=72)		
		Participants, n (%)	Score, mean (SD)	Change in score, mean (SD)	Participants, n (%)		Score, mean (SD)	Change in score, mean (SD)	Participants, n (%)		Score, mean (SD)	Change in score, mean (SD)
**DSRS-C^c^**												
	0	67 (99)	8.43 (5.51)	N/A^d^	70 (99)		9.26 (6.46)	N/A	72 (100)		11.21 (5.97)	N/A
	1	66 (97)	7.18 (5.34)	−1.18 (3.18)	69 (97)		8.12 (5.60)	−1.10 (4.11)	72 (100)		11.20 (6.03)	−0.01 (4.49)
	2	66 (97)	8.12 (6.04)	−0.29 (4.05)	68 (96)		9.46 (6.85)	0.25 (4.58)	72 (100)		10.54 (6.80)	−0.46 (3.98)
	4	66 (97)	7.40 (6.10)	−1.08 (4.11)	66 (93)		9.14 (6.68)	−0.02 (4.37)	70 (97)		10.76 (6.81)	−0.17 (4.81)
**AHP−SF^e^**												
	0	67 (99)	71.11 (13.38)	N/A	70 (99)		72.24 (12.43)	N/A	72 (100)		68.30 (15.30)	N/A
	1	66 (97)	71.05 (14.73)	0.34 (9.79)	69 (97)		73.14 (13.97)	1.14 (8.72)	72 (100)		69.30 (14.59)	0.94 (7.76)
	2	66 (97)	74.02 (14.39)	3.28 (9.85)	68 (96)		74.64 (13.54)	1.97 (9.75)	72 (100)		69.55 (15.79)	0.88 (8.32)
	4	66 (97)	75.13 (15.61)	3.93 (11.97)	66 (93)		75.94 (14.08)	2.98 (9.63)	70 (97)		70.60 (16.71)	2.61 (9.32)
**RSES^f^**												
	0	67 (99)	28.62 (5.90)	N/A	70 (99)		27.44 (5.56)	N/A	72 (100)		26.39 (6.48)	N/A
	1	66 (97)	29.75 (5.58)	1.03 (3.17)	68 (96)		28.54 (4.88)	1.21 (2.70)	72 (100)		26.77 (6.39)	0.32 (3.31)
	2	66 (97)	29.29 (6.48)	0.58 (3.22)	68 (96)		28.71 (5.95)	1.17 (3.39)	72 (100)		26.89 (6.25)	0.47 (3.34)
	4	66 (97)	29.44 (6.22)	0.77 (2.78)	66 (93)		28.64 (6.43)	0.92 (3.64)	70 (97)		27.28 (5.90)	0.91 (3.91)
**PedsQL^g^**												
	0	67 (99)	90.88 (11.99)	N/A	70 (99)		89.65 (11.40)	N/A	72 (100)		85.30 (13.13)	N/A
	1	66 (97)	93.48 (9.18)	2.60 (8.75)	69 (97)		90.61 (9.49)	0.94 (7.19)	72 (100)		85.89 (13.27)	0.54 (8.32)
	2	66 (97)	91.17 (14.92)	0.37 (10.17)	68 (96)		90.82 (10.46)	0.98 (10.85)	72 (100)		86.82 (14.99)	0.96 (12.15)
	4	66 (97)	92.43 (14.08)	1.37 (11.10)	66 (93)		90.41 (12.42)	0.69 (12.59)	70 (97)		87.63 (13.83)	2.31 (9.66)

^a^WCV: well-care visit.

^b^CBT: cognitive behavioral therapy.

^c^DSRS-C: Depression Self-Rating Scale for Children.

^d^N/A: not applicable.

^e^AHP-SF: Adolescent Health Promotion Short Form.

^f^RSES: Rosenberg Self-Esteem Scale.

^g^PedsQL: Pediatric Quality of Life Inventory.

**Figure 3:**
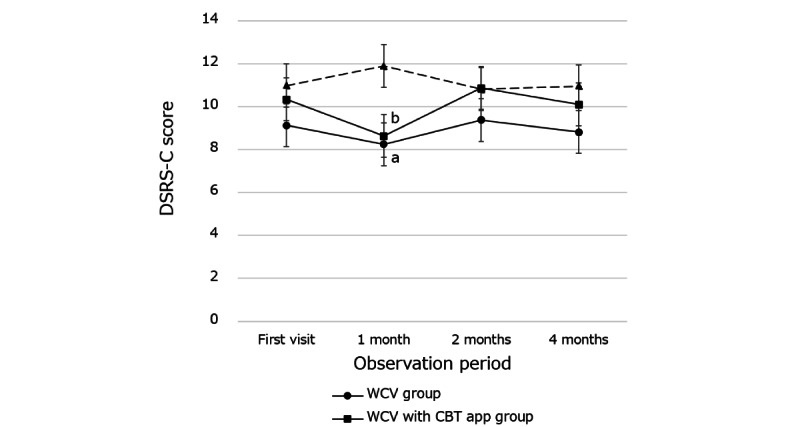
Changes in Depression Self-Rating Scale for Children (DSRS-C) scores in the intervention and nonintervention groups during the follow-up period; a: There were significant differences in the changes in DSRS-C scores from baseline to 1 month between the WCV group and the nonintervention group (*P*=.045); b: there were significant differences in the changes in DSRS-C scores from baseline to 1 month between the WCV with CBT app group and the nonintervention group (*P*=.004). Vertical bars show the SE. CBT: cognitive behavioral therapy; WCV: well-care visit.

No immediate or maintenance effects by intervention was observed. However, in high school students, there were significant differences in the changes in DSRS-C scores from baseline to 1 month between the WCV group (mean −0.88, SD 3.16) and the nonintervention group (mean 0.90, SD 4.49) and between the WCV with CBT app group (mean −1.67, SD 3.80) and the nonintervention group (Figure 3).

**Figure 4:**
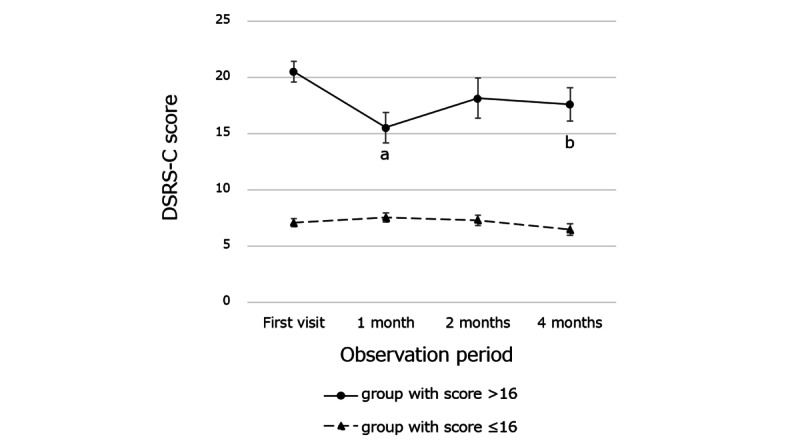
Changes in Depression Self-Rating Scale for Children (DSRS-C) scores for participants in the intervention groups by the DSRS-C cutoff score; a: the mean DSRS-C score for participants scoring >16 in the intervention groups was significantly decreased at 1 month compared with the mean score at their first visit (*P*=.004); b: the mean DSRS-C score for participants scoring >16 in the intervention groups was significantly decreased at 4 month compared with the mean score at their first visit (*P*=.03). Vertical bars show SE.

Furthermore, an intervention effect was observed in the classification of participants who scored above the DSRS-C cutoff score (16 points). The mean DSRS-C score for participants scoring >16 in the intervention groups was significantly decreased at 1 month (mean 15.56, SD 5.68) and at 4 months (mean 17.63, SD 5.88) compared with the mean score at their first visit (mean 20.53, SD 3.79). However, no such differences were observed in participants with DSRS-C score ≤16 in the intervention groups (Figure 4).

**Figure 5:**
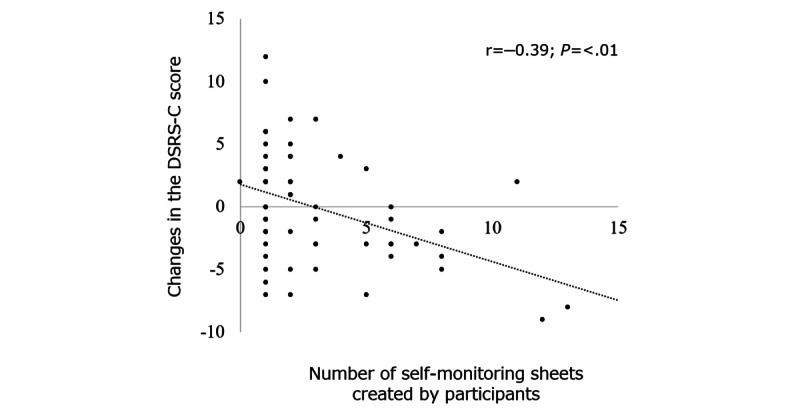
Correlation between the number of self-monitoring sheets created by participants and the changes in Depression Self-Rating Scale for Children (DSRS-C) scores in the well-care visit with cognitive behavioral therapy app group. Significant negative correlation was observed between the changes in DSRS-C scores and number of self-monitoring sheets created by participants at 4 months.

Regarding the effect of the CBT app on changes in the DSRS-C score, participants who created more self-monitoring sheets had better DSRS-C scores at the 4-month follow-up. The relationship between the number of self-monitoring sheets and the changes in DSRS-C score showed a significant negative correlation at 4 months (Figure 5).

### Secondary Outcome Result

#### AHP-SF Scores

**Figure 6:**
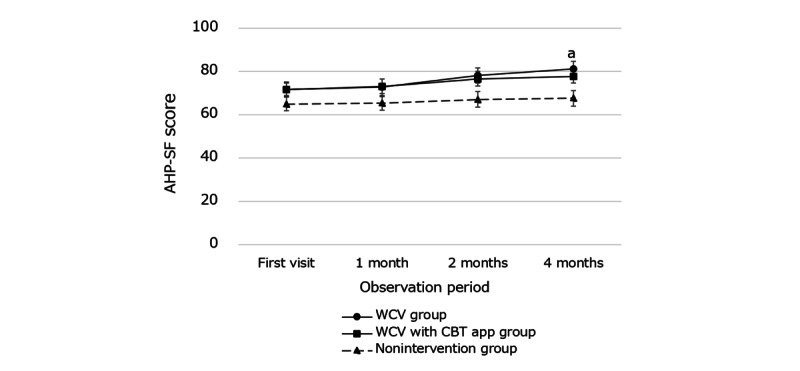
Changes in Adolescent Health Promotion Short Form (AHP-SF) scores in the intervention and nonintervention groups during the follow-up period; a: the changes in AHP-SF scores from baseline to 4 months were significantly different between the WCV group and the nonintervention group (*P*=.046). Vertical bars show the SE. CBT: cognitive behavioral therapy; WCV: well-care visit.

For all participants, the mean changes in AHP-SF scores from baseline to 1, 2, and 4 months did not significantly differ among the 3 groups (Table 1). However, in junior high school students, the changes in AHP-SF scores from baseline to 4 months were significantly different between the WCV group (mean 11.87, SD 19.06) and the nonintervention group (mean 3.33, SD 9.60; Figure 6).

#### RSES Scores

For all participants, the mean changes in RSES scores from baseline to 1, 2, and 4 months did not significantly differ among the 3 groups (Table 1). No significant difference was observed in junior high school students or high school students.

#### PedsQL Scores

For all participants, there was no significant difference in the mean changes in PedsQL scores from baseline to 1, 2, and 4 months among the 3 groups (Table 1). No significant difference was observed in junior high school students or high school students.

### Exploratory Outcome Measures

#### Suicidal Ideation

A total of 5.5% (12/217) of the participants presented with suicidal ideation during the observation period. Of these 12 participants, 6 (50%) participants were identified before entry, 5 (42%) participants were identified in the nonintervention group, and 1 (8%) participant was identified in the WCV with CBT app group. This showed a significant intervention effect for the prevention of suicidal ideation (Figure 1; *P*<.001).

#### Self-monitoring Effect of the CBT App

**Figure 7:**
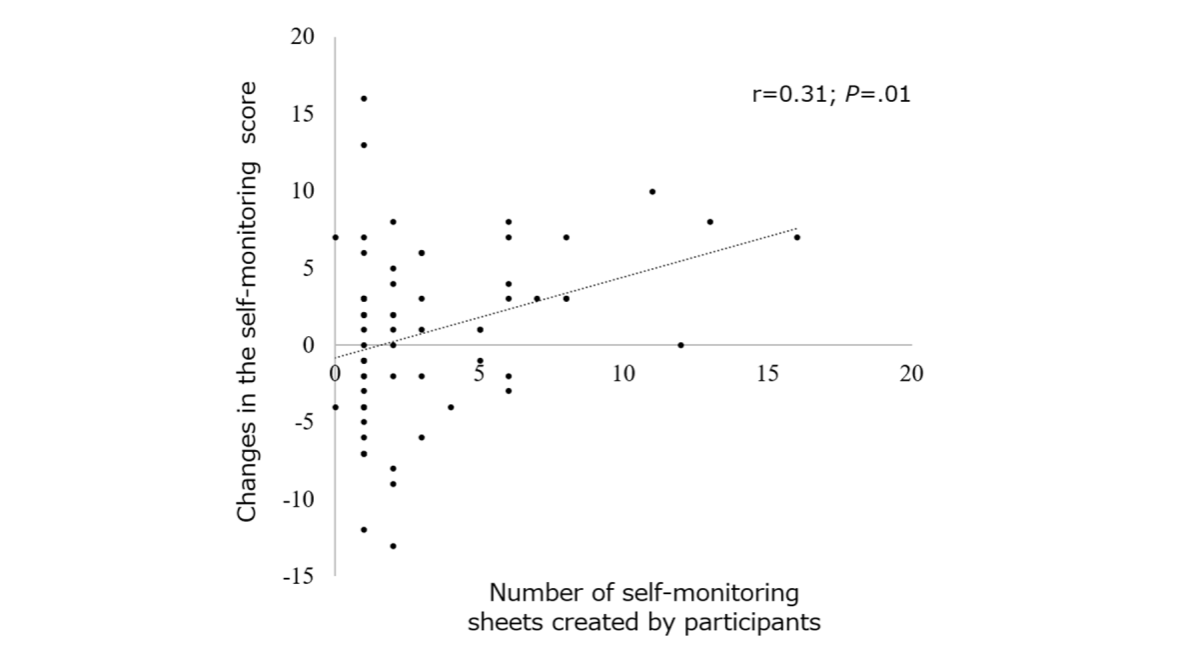
Correlation between the number of self-monitoring sheets created by participants and the changes in self-monitoring scores in the well-care visit with cognitive behavioral therapy app group. Significant positive correlation was observed between changes in self-monitoring scores and number of self-monitoring sheets created by participants at the 1-month visit.

Regarding the effect of the CBT app on changes in participants’ self-monitoring scores, the more participants created self-monitoring sheets, the better their self-monitoring scores were at the 1-month follow-up. The relationship between the number of self-monitoring sheets created by the participants and the changes in self-monitoring scores showed significant positive correlation (Figure 7).

## Discussion

### Principal Findings

In this randomized controlled trial, we were unable to demonstrate an effect of universal intervention with either the WCV intervention or the WCV with CBT app intervention in terms of changes in adolescents’ depressive symptoms; however, both interventions showed a temporary effect in improving depressive symptoms in high school students. Furthermore, the effect was significant for individuals who scored above the cutoff point for depressive symptoms. In addition, the interventions significantly reduced suicidal ideation during the observation period. However, our hypothesis of obtaining better outcomes in the WCV with CBT app group was not supported.

Initially, the interventions showed a significant temporary effect of improving depressive symptoms in high school students irrespective of intervention type (WCV only or WCV with CBT app). However, the second WCV session showed no effect on depressive symptoms in either intervention group after 1 month. This result may reflect volunteer bias, whereby participants’ responses met the expectations of the researchers. As volunteers who participate in research studies are generally high-functioning individuals with higher willingness [35,36], their responses tend to produce better results despite the intervention pattern. Although there was a possibility of volunteer bias, the intervention itself may have been effective for participants with a high level of depressive symptoms. The intervention effect was significant and prominent in participants who scored above the DSRS-C cutoff point (16 points) compared with those who scored ≤16. A similar intervention effect for adolescents using a CBT program was reported by Tomyn et al [33], where the intervention showed no average improvement in universal participants, except for those with elevated depression symptoms. This may mean that achieving improvements when participants have few depressive symptoms is challenging. However, our study indicated that better results may be obtained by targeting interventions to individuals with more depressive symptoms rather than a school-based universal intervention for all students with and those without depressive symptoms.

### Effect in Reducing Suicidal Ideation

This study revealed significant effect in terms of reduced suicidal ideation in adolescents during the 4-month observation period in both the WCV group and the WCV with CBT app group. Although 7% (5/72) of the participants were identified as having suicidal ideation in the nonintervention group during the study period, only 0.8% (1/132) of the participants in the intervention groups was identified as having suicidal ideation. This result suggested that the intervention may potentially be effective, and the WCV with or WCV without the CBT app may be an effective means to prevent children from committing suicide. As adolescent suicide is a global mental health concern [6,37], school-based universal prevention programs have focused on reducing the number of suicide attempts and suicidal ideation [37-42]. A European multicentral randomized controlled trial involving 11,110 adolescents (median age 15 years) from 168 schools showed that a short (5 hours over 4 weeks) school-based intervention including role-play sessions and interactive lectures about mental health was significantly effective in preventing new cases of suicide attempts and suicidal ideation at the 12-month follow-up [37]. The study accounted for its significant effect by the role-play sessions and interactive lectures, providing adolescents with opportunity to think, verbalize, and discuss a range of issues related to mental health. Checking social determinants of health and providing anticipatory guidance through the HEEADSSS-based interviews, as used in our study, may have offered adolescents the opportunity to identify their emotions and feelings. Therefore, their suicidal ideation may have been suppressed compared with those in the nonintervention group.

### Effect of a Smartphone CBT App

We developed a smartphone CBT app for adolescents, which contained psychoeducation and self-monitoring and was expected to improve their depressive symptoms. App users were coached to observe their own thoughts, feelings, body response, actions, and relationships relating to daily events by repeatedly creating monitoring sheets, and they could monitor their own mind and develop solutions for changing their cognitive processes throughout the sessions. The CBT app was significantly effective in terms of obtaining self-monitoring skills and reducing depressive symptoms, which was confirmed by the association between the number of self-monitoring sheets created and the changes in self-monitoring and depressive symptoms scores. This effect may have contributed to the suppression of the adolescents’ suicidal ideation similar to the WCV. An increasing number of mobile apps are available for adolescents with mental health problems, many of which are equipped with CBT programs. However, currently, there is insufficient research evidence to support the effectiveness of these apps for adolescents [17,18]. Stallard et al [16] developed a smartphone app that provided a personalized toolbox of strategies based on CBT in conjunction with a face-to-face intervention to reduce self-harm and support psychological functioning. They found that 73% of individuals who had recently harmed themselves reported reductions in self-harm and depressive scores; however, a flaw in their study design was the absence of a comparison group. Few randomized controlled trials have focused on smartphone apps for adolescents’ mental health, and most available studies have failed to demonstrate significant effects on the intended outcomes [43]. Although our study was designed as a randomized controlled trial, both intervention groups included face-to-face interview (WCV) and our hypothesis of obtaining better outcomes in individuals who receive both WCV with CBT app was not supported. Thus, more scientific evidence for the significance of CBT apps is needed from future research. However, our finding of a significant association between the number of self-monitoring sheets created and the changes in self-monitoring scores and depressive symptoms offered a further perspective of implementation of apps for adolescent mental health services. In addition, a deep learning approach using text mining data created in the 5 window panels of the smartphone app in this study could help health care professionals to find adolescents in need of medical care at an advantage.

### Effect of HEEADSSS

Another important finding in this study was the significant increase in health promotion scale scores at the 4-month follow-up in junior high school students in the WCV group. This indicated that they may have become interested in health promotion activities, such as nutrition, exercise, and stress management. Participants in the WCV group participated in risk assessment interviews and received counseling (HEEADSSS) twice (at baseline and 1 month after the first visit). Although several school-based interventions to promote adolescent health revealed both significant and nonsignificant effects in reducing health problems [37,38,40,41], no evidence of effectiveness was available for individual interventions in primary care settings. Our WCV with a HEEADSSS-based interview allocated sufficient intervention time (>30 minutes), which enabled participants to talk and think about their own health through the HEEADSSS framework. As the HEEADSSS framework in a face-to-face interview requires time, an electronic HEEADSSS screening system has been widely accepted [44]. Although annual health checkup for adolescents in Japan have been performed at each school by the school physicians under the supervision of the Ministry of Education of Japan, the school health examination only includes a physical examination (eg, measuring height and body weight; checking visual acuity, hearing, and scoliosis; and urinalysis). Therefore, screening and preventing mental health problems using a HEEADSSS-based interview is required at primary care clinics.

### Limitations

This study had some limitations that need to be addressed. First, the follow-up period in this study (4 months) was a relatively short observation period to draw conclusions about a universal intervention effect. Although there were significant differences in the prevalence of suicidal ideation between the intervention and nonintervention groups during the observation period, participants may have developed suicidal ideation after the observation period, even in the intervention groups. However, many adolescent intervention studies have used relatively short assessment durations (eg, 4-12 weeks) [13,15,34]. As adolescents’ mental health conditions may easily change based on daily events, regular additional interventions may be necessary to obtain significant outcomes in longer observation periods. Second, although this study found significant associations between the number of self-monitoring sheets created and improvement in depressive symptoms and self-monitoring skills, a revised version of the CBT app is required to enhance feasibility and adherence. Many participants in the WCV with CBT app group created only a couple of self-monitoring sheets during the observation period, and the efficacy of the CBT app may be enhanced if they could be challenged to make more self-monitoring sheets using an additional method such as gamification [45,46]. Furthermore, our CBT app consisted of two modules (psychoeducation and self-monitoring), and additional modules including cognitive restructuring and behavioral activation modules are necessary to increase the strength of CBT. Finally, although the 217 participants in this study were from 48 junior high schools and high schools, which could avoid the bias caused by sharing CBT app information in single school [32], there was a possibility of volunteer bias because highly motivated participants may have been more interested in participating. Therefore, we should plan to implement a further school-based intervention study.

### Conclusions

In conclusion, this study contributes by informing research directions to promote adolescent health. A standard interview framework for adolescent health promotion (ie, HEEADSSS) may be applied in primary care settings in Japan to improve adolescents’ mental health, as there are no screening and intervention systems in the regular school-based health checkup. To minimize the time required for this screening, development of either a short form of the HEEADSSS or electronic HEEADSSS screening may be required. Furthermore, our CBT app, which uses a mobile device, may emerge as a new health promotion tool for adolescents if more CBT modules are added. Integrating direct and indirect interventions (HEEADSSS and CBT apps, respectively) may further promote adolescent health.

## Appendix

Multimedia Appendix 1 CONSORT e-HEALTH checklist (V 1.6.1).

## Abbreviations

AHP-SF: Adolescent Health Promotion Short Form
CBT: cognitive behavioral therapy
DSRS-C: Depression Self-Rating Scale for Children
HEEADSSS: Home, Education, Eating, Activities, Drugs, Sexuality, Suicide, Safety
MDD: major depression disorder
PedsQL: Pediatric Quality of Life Inventory
PHQ-9: 9-item Patient Health Questionnaire
RSES: Rosenberg Self-Esteem Scale
TEIQue-ASF: Trait Emotional Intelligence Questionnaire–Adolescent Short Form
WCV: well-care visit
